# A new variety of chondrocoracoideus muscle, or an additional head of pectoralis major muscle

**DOI:** 10.1007/s00276-022-02887-x

**Published:** 2022-01-21

**Authors:** Nicol Zielinska, Kacper Ruzik, Georgi P. Georgiev, Iva N. Dimitrova, R. Shane Tubbs, Łukasz Olewnik

**Affiliations:** 1 Department of Anatomical Dissection and Donation, Medical University of Lodz, Lodz, Poland; 2 Department of Orthopedics and Traumatology, University Hospital Queen Giovanna-ISUL, Medical University of Sofia, Sofia, Bulgaria; 3 Department of Anatomical Sciences, St. George’s University, True Blue, Grenada; 4 Department of Neurosurgery, Tulane University School of Medicine, New Orleans, LA USA; 5 Department of Neurology, Tulane University School of Medicine, New Orleans, LA USA; 6 Department of Structural and Cellular Biology, Tulane University School of Medicine, New Orleans, LA USA; 7 Department of Surgery, Tulane University School of Medicine, New Orleans, LA USA; 8 Department of Neurosurgery, Ochsner Medical Center, New Orleans, LA USA

**Keywords:** Pectoralis major muscle, Pectoralis minor muscle, Chondrocoracoideus, Accessory muscle, Morphological variation, Thoracic outlet syndrome

## Abstract

The pectoralis major and pectoralis minor muscles are located in the anterior chest wall. This region is characterized by high morphological variability. During dissection an additional muscle was found, originating from the lateral border of the pectoralis major muscle. After fusion it passed into the tendinous part coursing under the insertion of the pectoralis major muscle, then formed a common junction with the short head of the biceps brachii muscle, the distal attachment of which is on the coracoid process. Such an accessory structure could lead to neurovascular compression and cause thoracic outlet syndrome, of which pain is usually the first symptom. This muscle has not been described in the literature so far and for that reason we can name the present case as an unique structure.

## Introduction

The pectoralis region is characterized by high morphological variability. Interestingly, the PM or PMi can be completely absent [1]. On the other hand, there are cases in which the PM is doubled. Some accessory structures can also occur. For example, the pectoralis quartus originates near the costochondral junction of the fifth and sixth ribs and inserts into the axillary arch or sternalis muscle (this is also a morphological variation, found in only 3–5% of the population). We can also distinguish the pectoralis intermedius, pectoralis minimus, chondrofascialis, sternohumeralis and sternochondrocoracoideus muscles [1].

The present report describes an accessory structure originating in the muscle belly of the PM and fusing with the lateral border of the PM at the level of the seventh rib. After this fusion it passes into tendinous part, crossing under the PM, and is then connected to the short head of the biceps brachii; the common junction has its insertion on the coracoid process. Knowledge of the morphological variability of this muscle is essential for all clinicians.

## Case report

**Fig. 1:**
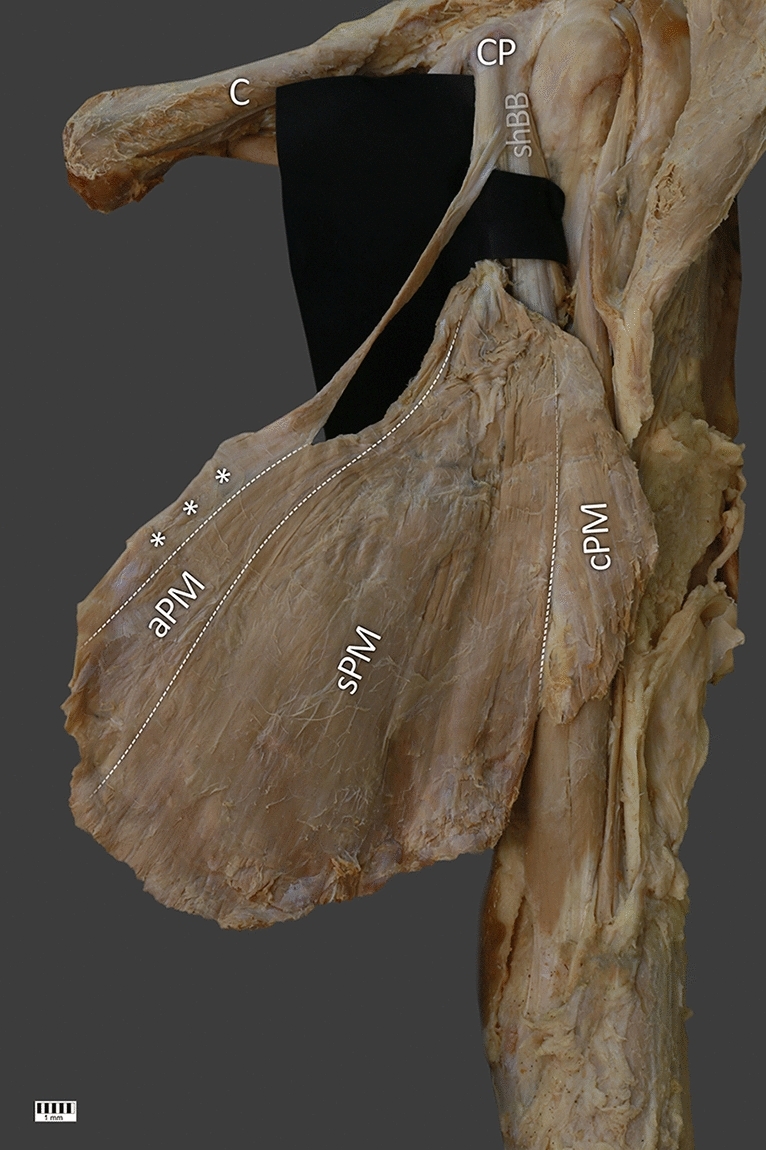
Anterior view of the rare case of the pectoralis major muscle. C clavicle, cPM clavicular part of the pectoralis major muscle, sPM sterno-costal part of the pectoralis major muscle, aPM abdominal part of the pectoralis major muscle, CP coracoid process, shBB short head of the biceps brachii muscle. lhBB long head of the biceps brachii muscle, the white stars indicates the accessory part of the pectoralis major muscle

A 78-year-old cadaver (male) at death was subjected to routine anatomical dissection for research and teaching purposes at the Medical University of Lodz in Poland. The chest and left upper limb region underwent traditional anatomical dissection [2], fixed in 10% formalin solution. Dissection began with removal of the skin, superficial fascia, and fat tissue from the area of the shoulder, the medial side of the arm, and anterior part of the chest. The next step included visualizing the deltoid muscle and the PM, and accurate visualization of the biceps brachii and the coracobrachialis muscles. Following this, all structures were thoroughly cleaned, and an additional pectoral muscle was found—Fig. 1.

In the present case, the PM was divided into three parts: clavicular, sternocostal, and abdominal. The length of the sternocostal part, measured to the passage into the tendinous attachment, was 180.23 mm. The width of this part was 139.62 mm. The tendinous attachment on the lateral lip of the bicipital groove of the humerus was fused with the tendinous portion of the clavicular part, which was 127.38 long. The width, 19.09 mm, was measured from the point of an origin on the medial half of the clavicle to the line connecting the clavicular and sternocostal parts. The third, abdominal, part was 164.44 mm long and 15.99 mm wide—Table 1.

**Table 1:** Morphometric measurements of distinct parts of the PM and the accessory muscle

	Sternocostal part (mm)	Clavicular part (mm)	Abdominal part (mm)
Length	180.23	127.38	164.44
Width	139.62	19.09	15.99

	Accessory pectoral muscle (mm)
Length	201.88
Muscular part	102.96
Tendinous part*	72.96
Common junction	25.96
Width**	8.76

*To the connection with the short head of the biceps brachii

**In the point of the origin

**Fig. 2:**
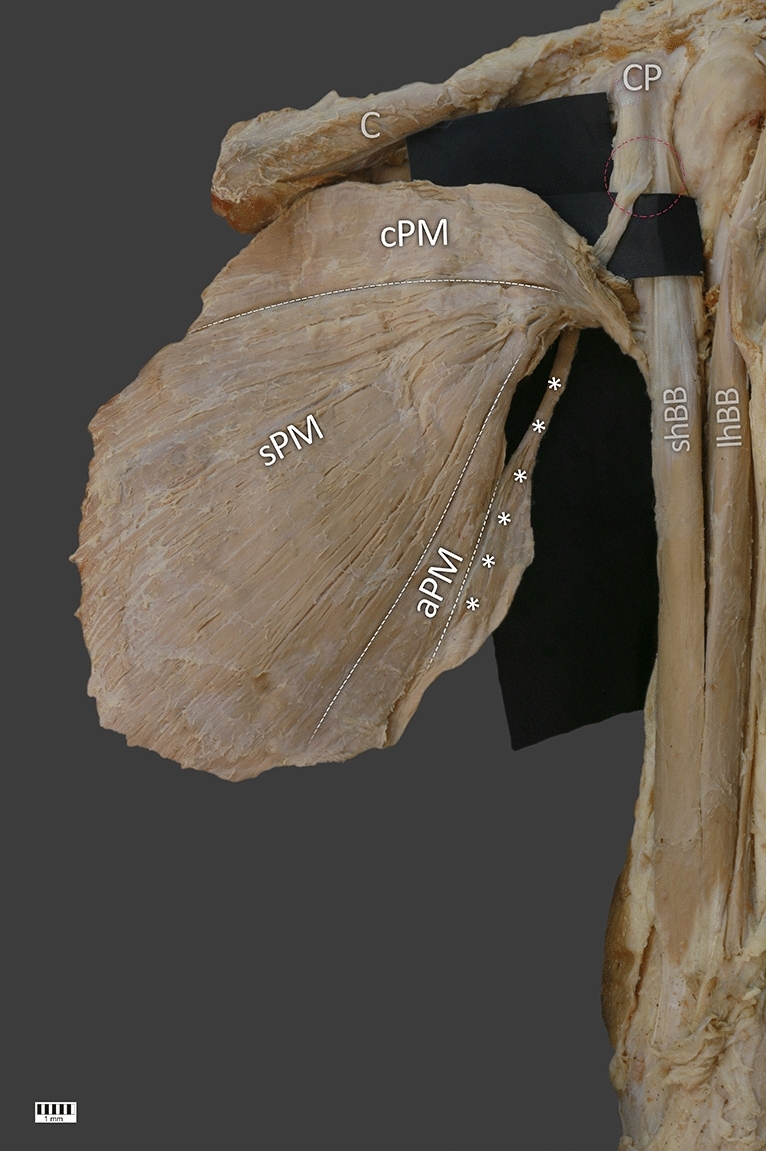
Posterior view of the rare case of the pectoralis major muscle. C clavicle, cPM clavicular part of the pectoralis major muscle, sPM sterno-costal part of the pectoralis major muscle, aPM abdominal part of the pectoralis major muscle, CP coracoid process, shBB short head of the biceps brachii muscle, the white stars indicates the accessory part of the pectoralis major muscle

During this anatomical dissection an additional structure divided into muscle belly and tendon was found. Its muscular origin was fused with the lateral border of the muscular abdominal part of the PM at the level of the seventh rib. The length of this fragment was 102.96 mm. At the point of origin the width was 8.76 mm. Next, it passed into a tendinous structure crossing the PM under its tendinous distal attachment on the lateral lip of the bicipital groove of the humerus. Its length measured from the myotendinous junction to the connection with the short head of the biceps brachii was 72.96 mm. The common junction, 25.96 mm long, was attached to the coracoid process—Figs. 1, 2.

An electronic caliper (Mitutoyo Corporation, Kawasaki-shi, Kanagawa, Japan) was used for the measurements. Each measurement was repeated twice with an accuracy of up to 0.1 mm.

No other morphological variabilities were found. Table 1 shows the morphometric measurements of the presented case.

## Discussion

As mentioned above, there are several morphological variations in the thoracic region. Knowledge of them could help us to assign an appropriate name to the newly found muscle.

The first interesting variant is a pectoralis minimus muscle, also known as the sternocostocoracoidian. It originates from the first rib cartilage and its insertion is on the coracoid process. In one study, the pectoralis minimus was observed in 5.35% of the population [3]. All cases were innervated by the lateral pectoral nerves [3].

Another morphological variation is a pectoralis intermedius, which originates on the third and fourth ribs between the PM and PMi. It is usually inserted on the coracoid process [1]. The pectoralis intermedius usually coexists with the pectoralis quartus, its proximal attachment arising near the costochondral junction of the fifth and sixth ribs [4]. It inserts to the PM [1].

Another variation is the chondroepitrochlearis, otherwise named costoepitrochlearis or chondrohumeralis, originating from one or more ribs and inserting on to the medial epicondyle of the humerus or into the median intermuscular septum [5]. Some authors found the same muscle but originating directly from the PM [6, 7].

The chondrocoracoideus muscle, sometimes called the costocoracoideus or muscle of Wood [8], is another rare variant of the PM. It originates from the sixth to the eighth ribs and the rectus sheath. Its distal attachment is connected to the short head of the biceps brachii and jointly attached to the coracoid process [1].

Venieratos et al. [9] found a PM of which the abdominal portion was the accessory pectoral muscle originating in three slips from the sixth to eighth ribs and the external oblique muscle aponeurosis. It was distally fused with the short head of the biceps brachii and then attached to the coracoid process. They called this accessory structure the chondrocoracoideus muscle.

Douvetzemis et al. [10] described a similar case emerging as three slips from the sixth to eighth ribs and the external oblique muscle aponeurosis. At the level of the fifth and sixth ribs it was connected to the sternocostal part of the PM. Its insertion was on the coracoid process, but before the attachment was a small fusion with the short head of the biceps brachii [10]. Tubbs et al. [11] described a PM with an insertion into the shoulder joint capsule.

Considering the foregoing information, the additional muscle in the present case seems most similar to the chondrocoracoideus muscle. However, there are differences indicating that it is not the chondrocoracoideus, but an additional head of the PM or a distinct muscle that has not been described in the literature so far. First, the proximal attachment was represented as one muscle belly not three distinct slips as in the cases described by Douvetzemis et al. [10] and Venieratos et al. [9] Second, the muscle in our case originated from the lateral border of the PM as a fusion with the abdominal part of the PM at the level of the seventh rib. In the cases found by the aforementioned workers, the proximal attachment created the abdominal portion of the PM originating from the sternal line at the level of the sixth to eighth ribs. Clinically, the important fact seems to be that the tendinous part of the accessory muscle in our case was located deeper to the tendinous part of the PM and passed under this muscle. In the cases described by Douvetzemis et al. [10] and Venieratos et al. [9] the distal attachment connected to the short head of the biceps brachii did not interfere with the insertion of the PM on the lateral lip of the bicipital groove of the humerus. These differences suggest it would be wrong to call this new structure the chondrocoracoideus. It would be more appropriate to define it as an extra head of the PM, or to find a new name.

Most of the various additional structures in this region are implicated in neurovascular compression. The present accessory muscle could potentially compress the axillary artery and branches of the brachial plexus. It could lead to thoracic outlet syndrome, which has a wide range of symptoms [5].

Compression of the vein (which is also possible) could lead to swelling in the upper limb region, pain, venous distention, and—more dangerously—deep venous thrombosis. When the artery is compressed, the results include a bluish discoloration of the limb and a faint pulse. However, the most common cause of thoracic outlet syndrome is entrapment of the brachial plexus branches, and pain is usually the first symptom. Of course there can be other pathologies such as atrophy of the muscles innervated by the compressed nerve or loss of sensation or impaired movement [12].

Moreover, the specific course of the present accessory muscle could increase the likelihood of neuropathy. The tendinous part passed under the distal attachment of the PM, so if some nerve branches were located between the two structures, the symptoms found in every neuropathy could result.

Although the additional structure in the present case could result in pathologies, it could also have advantages. For example, it could be used in such surgeries as breast, axilla or glenohumeral joint reconstruction. Van de Sande et al. [13] described a surgery in which a flat slip of the PMi was used to reconstruct the glenohumeral joint. It depended on suturing to the anterior labrum and the bicipital groove using bone tunnels [13]. However, using the PMi for such an operation can impair the function of this muscle, potentially causing weakness in the lowering and abduction of the upper limb girdle and destabilization of the scapula [14]. Therefore, it seems better to use the accessory muscle than the PMi for reconstruction, because there are no such side effects.

For the purpose of surgeries, it is helpful to know all possible morphological variations in the thoracic region. Without this knowledge, some results of computed tomography or magnetic resonance showing additional structures could confuse a surgeon. It is worse if the change is detected during the operation; it could cause complications or prolong the surgery. The ability to recognize morphological variation allows surgeons to be better prepared for performing axillary lymphadenectomy or mastectomy.

The wondering thing was if the present case is a new variety of chondrocoracoideus muscle, or an additional head of the PM. Considering the foregoing information, the additional muscle in the present case seems most similar to the chondrocoracoideus muscle. However, there are differences indicating that it is not the chondrocoracoideus, but an additional head of the PM or a distinct muscle that has not been described in the literature so far and for that reason we can name the present case as an unique structure. As it mentioned above, it is potentially associated with neurovascular compression which may result in neuropathy, pain, or deep venous thrombosis. There are also positive aspects of this muscle, like using it as a material for breast reconstruction.

## Conclusion

The pectoral region is highly morphologically variable. Although the course of the muscle in the present case is similar to that described for the chondrocoracoideus, there are some differences. This additional muscle could cause thoracic outlet syndrome via neurovascular compression. On the other hand, it could be used during reconstructive surgeries, so knowledge of it is essential not only for anatomists but also for clinicians.

## Data Availability

Please contact authors for data requests (Łukasz Olewnik, PhD—email address: lukasz.olewnik@umed.lodz.pl).
